# Gustave Roussy immune score as an independent prognostic factor for treatment response and survival in advanced renal cell carcinoma treated with nivolumab in second-line and beyond

**DOI:** 10.3389/fonc.2025.1657053

**Published:** 2025-10-16

**Authors:** Bülent Erdoğan, İvo Gökmen, Cagnur Elpen Kodaz, Ahmet Küçükarda, Didem Divriklioğlu, İsmail Bayrakçı, İlhan Hacıbekiroğlu, Esra Özen Engin, Muhammet Bekir Hacıoğlu, Erkan Özcan, Gökhan Öztürk, Hilmi Kodaz

**Affiliations:** ^1^ Medical Oncology, Trakya Universitesi Tip Fakultesi, Edirne, Türkiye; ^2^ Family Medicine, Bilecik Seyh Edebali Universitesi, Bilecik, Türkiye; ^3^ Medical Oncology, Sakarya Universitesi Tip Fakultesi, Sakarya, Türkiye; ^4^ Department of Medical Oncology, Acıbadem Eskişehir Hospital, Eskişehir, Türkiye

**Keywords:** metastatic renal cell carcinoma, GRIm score, nivolumab, second-line therapy, prognostic biomarker

## Abstract

**Background:**

Metastatic renal cell carcinoma (mRCC) represents a significant challenge due to variable patient outcomes despite advancements in treatment. Nivolumab, a programmed death-1 (PD-1) inhibitor, has demonstrated efficacy as a second-line or later therapy following progression on tyrosine kinase inhibitors (TKIs). However, identifying reliable prognostic biomarkers remains critical. The Gustave Roussy Immune (GRIm) score, incorporating serum albumin, lactate dehydrogenase (LDH), and neutrophil-to-lymphocyte ratio (NLR), may provide prognostic value in this context.

**Methods:**

This multicenter retrospective cohort study included 81 mRCC patients treated with nivolumab as second-line or subsequent therapy following progression on first-line TKIs (e.g., sunitinib or pazopanib). Patients were categorized into low (0–1) and high (2–3) GRIm score groups based on pre-treatment laboratory values. Outcomes included progression-free survival (PFS), overall survival (OS), and treatment response, assessed using RECIST criteria. Survival analyses were performed using Kaplan-Meier curves, and prognostic factors were identified through univariate and multivariate analyses.

**Results:**

The median age was 63 years, and 72.8% were male. Patients with low GRIm scores demonstrated significantly higher objective response rates (44.4% *vs*. 11.1%; p = 0.01) and longer OS (23.3 *vs*. 8.8 months; p = 0.004). PFS was also significantly longer in the low GRIm score group (8.7 *vs*. 3.1 months; p = 0.015). Multivariate analysis identified a high GRIm score as an independent predictor of worse OS (HR: 0.46; p = 0.03).

**Conclusion:**

The GRIm score effectively stratifies mRCC patients treated with nivolumab, identifying those with significantly better survival and treatment responses. As a simple, cost-effective tool, it offers potential for integration into clinical practice to guide personalized treatment strategies. Further prospective studies are warranted to validate these findings.

## Introduction

The most common kidney cancer in adults is renal cell carcinoma (RCC), accounting for approximately 2–3% of all cancers (1). Despite significant therapeutic advances, metastatic RCC (mRCC) remains a significant cause of cancer-related death due to its high fatality rates. Over the past decade, substantial progress has been made in the treatment of mRCC. Traditional cytokine-based therapies have been replaced by targeted agents, including multitargeted tyrosine kinase inhibitors targeting angiogenesis (VEGFR-TKIs) and mammalian target of rapamycin (mTOR) inhibitors (2). Immune checkpoint inhibitors (ICIs) have recently revolutionized mRCC management by reactivating T-cell-mediated antitumor immunity (3, 4).

In 2015, nivolumab became the first ICI approved for mRCC following the CheckMate-025 trial, which demonstrated its survival benefit over everolimus in patients resistant or intolerant to VEGFR-TKIs (5). Currently, ICI-based combinations have become the standard first-line therapy across all risk groups defined by the International Metastatic RCC Database Consortium (IMDC), with particular efficacy in intermediate- and poor-risk patients (6, 7). However, despite these advancements, nivolumab continues to be a pivotal second-line treatment option following VEGFR-TKI therapy (5).

While ICIs have significantly improved outcomes for many patients, accurately identifying those most likely to benefit remains a challenge. Prognostic models, such as the Memorial Sloan Kettering Cancer Center (MSKCC) and IMDC scoring systems, are widely used to stratify mRCC patients (7, 8). However, these models were developed based on patients treated with cytokines or VEGFR-TKIs, limiting their relevance in the era of immunotherapy. This underscores the critical need for novel and effective prognostic tools specifically tailored to ICI-treated mRCC patients.

Cancer-associated inflammation plays a crucial role in prognosis and therapeutic responses. Several inflammatory indices derived from peripheral blood, including neutrophil-to-lymphocyte ratio (NLR) (9–11), platelet-to-lymphocyte ratio (PLR) (10–12), derived NLR (10), systemic inflammation index (SII) (10, 11), and others, have shown potential in stratifying patients receiving ICIs, including nivolumab. However, despite their promising results, none of these indices have transitioned into routine clinical practice.

In this context, the Gustave Roussy Immune (GRIm) score represents a potential candidate for clinical implementation. Developed by Bigot et al. during phase I immunotherapy trials, the GRIm score was specifically designed to stratify patients undergoing immune checkpoint inhibitor therapy (13). By integrating albumin, lactate dehydrogenase (LDH), and NLR, the GRIm score provides a practical, cost-effective approach to patient prognostication. Its utility has been demonstrated in lung and gastrointestinal cancers, where it has shown promising results in guiding treatment decisions. However, its relevance in RCC, particularly in patients treated with nivolumab, has not yet been explored (14). Given its origin in immunotherapy and its focus on inflammatory markers, the GRIm score may address the unmet need for a practical prognostic tool in this setting.

This study aims to assess the prognostic value of the GRIm score in mRCC patients receiving nivolumab. By exploring its utility, we aim to determine whether the GRIm score can bridge the gap in prognostic stratification for ICI-treated mRCC patients, potentially facilitating its integration into clinical practice.

## Materials and methods

This study was approved by the Ethics Committee of Trakya University Faculty of Medicine (Registration Number: TUTF-GOBAEK 2024/436) and conducted at two centers: the Medical Oncology Clinics of Trakya University and Sakarya University Faculties of Medicine. It was carried out in accordance with the Declaration of Helsinki, Good Clinical Practice, and local ethical guidelines, ensuring full compliance with ethical standards.

### Study population and study design

This multicenter retrospective study analyzed clinical, pathological, and radiological data, along with laboratory parameters, from patients with mRCC treated with nivolumab in a real-world setting. Data were retrieved from medical records across participating centers. The study covered a 10-year period from 2014 to 2024 and included 81 patients who met the following criteria: age older than 18 years, cytologically or histologically diagnosed mRCC, at least one infusion of nivolumab administered as second-line or later therapy during standard clinical practice, and prior treatment with at least one anti-VEGF therapy during the metastatic phase. Patients were excluded if they had coexisting hematological disorders, severe systemic infections, documented renal or hepatic insufficiency, or a history of prior immunotherapy.

### Laboratory data and GRIm score assessment

Among the clinical and pathological data collected, laboratory parameters held particular importance due to their role in determining the GRIm score. Laboratory assessments included LDH and albumin levels, measured using automated chemistry analyzers (e.g., Roche Hitachi Cobas 8000, Rotkreuz, Switzerland), as well as neutrophil and lymphocyte counts, obtained through hematology analyzers (e.g., Sysmex SE-9000, Kobe, Japan).

To ensure the relevance of baseline values, laboratory parameters were measured within 14 days prior to the initiation of nivolumab treatment. The GRIm score was calculated for each patient using the following criteria:

- LDH levels: within the normal range (0 points), above the upper normal limit (UNL) (1 point). The UNL for LDH was defined as 200 U/L at both participating centers.
- Albumin levels: ≥ 35 g/L (0 points), < 35 g/L (1 point).
- NLR: ≤ 6 (0 points), > 6 (1 point).

Patients were stratified into two risk categories according to their GRIm scores: low-risk (scores of 0–1) and high-risk (scores of 2–3).

### Treatment protocol and follow-up procedures

Nivolumab was administered intravenously at a dose of either 3 mg/kg or a fixed dose of 240 mg every two weeks, depending on clinical practice guidelines. Treatment was continued until disease progression, unacceptable toxicity, death, or patient preference. In cases where patients demonstrated clinical benefit despite radiological disease progression, therapy was allowed to continue based on the discretion of the treating physician.

Patients were monitored from the initiation of nivolumab therapy until death or the date of their last recorded clinical evaluation. Disease progression was assessed using advanced imaging modalities, including contrast-enhanced positron emission tomography–computed tomography (PET-CT), computed tomography (CT), and cranial magnetic resonance imaging (MRI). Imaging evaluations were typically performed at intervals of 6 to 12 weeks, depending on the clinical judgment of the physician and the disease status.

The study follow-up period concluded in September 2024, ensuring a robust observation window for analyzing treatment outcomes and progression patterns in the real-world setting.

### Clinical outcomes and endpoints

The primary endpoints of the study were progression-free survival (PFS) and overall survival (OS). PFS was defined as the time from the first nivolumab administration to disease progression (radiological or clinical) or death from any cause, censored at the last follow-up for patients alive without progression. OS was calculated from initiation of nivolumab to death from any cause or censoring at the last follow-up for patients alive or lost to follow-up.

Secondary endpoints included objective response rate (ORR), disease control rate (DCR), and duration of response (DOR). Tumor responses were categorized as complete response (CR), partial response (PR), stable disease (SD), or progressive disease (PD) according to the Response Evaluation Criteria in Solid Tumors (RECIST). ORR was defined as the sum of CR and PR, while DCR included CR, PR, and SD. DOR was calculated from the date of CR or PR to progression or death, censored at the last follow-up for patients alive without progression.

These endpoints provided a comprehensive evaluation of nivolumab’s clinical efficacy in real-world mRCC management.

### Statistical analysis

Patient demographic and clinical characteristics were summarized using descriptive statistics. Categorical variables were presented as frequencies and percentages, while continuous variables were expressed as medians with interquartile ranges (IQR). Differences in demographic and clinical variables between low- and high-risk GRIm score groups were analyzed using chi-square or Fisher’s exact tests, as appropriate.

Survival outcomes, including PFS, OS, and DOR, were estimated using the Kaplan-Meier method, and differences between groups were compared using the log-rank test.

Variables with a p-value < 0.05 in univariable analysis and well-known prognostic factors were included in multivariable models. Univariable and multivariable analyses were conducted using Cox proportional hazards regression models to calculate hazard ratios (HR) and 95% confidence intervals (CI). Statistical significance was set at p < 0.05 for all analyses. All statistical analyses were performed using IBM SPSS Statistics version 23.0 (IBM Corp., Armonk, NY, USA).

## Results

### Baseline patient characteristics and comparison of GRIm score groups

A total of 103 patients were retrieved, but 81 patients fulfilling inclusion and exclusion criteria were included in this study, with a median age of 63 years (IQR: 56–70.5). The majority were male (72.8%), and 84% had clear-cell carcinoma histologically. In terms of treatment lines, 48.1% of patients received nivolumab as second-line therapy, while 51.9% were treated beyond the second line. ECOG performance status indicated that 61.7% of patients were categorized as ECOG 0, while 38.3% had an ECOG score of ≥1.

According to the IMDC risk classification, 23.5% of patients were in the favorable risk group, 61.7% in the intermediate risk group, and 14.8% in the poor risk group. Similarly, MSKCC scoring categorized 19.8% as good risk, 61.7% as intermediate risk, and 18.5% as poor risk. Regarding metastasis distribution, 46.9% of patients had bone metastases, 17.3% had lung metastases, and 19.8% had liver metastases.

When comparing GRIm score groups, no significant differences were observed in demographic, clinical, or most metastatic characteristics between the low GRIm score group (n = 63) and the high GRIm score group (n = 18), except for lung metastases, which were significantly more common in the low GRIm score group (p = 0.04). Additionally, patients in the high GRIm score group were more likely to receive nivolumab in later treatment lines, though this difference did not reach statistical significance (p = 0.08). Trends toward worse ECOG performance status (p = 0.09) and higher IMDC risk scores (p = 0.10) were also observed in the high GRIm score group. These findings are summarized in **Table 1**.

**Table 1:** Distribution of demographic and clinical characteristics across the entire cohort and GRIm score groups.

Parameters	All patients (n = 81)	GRIm low group (n = 63)	GRIm high group (n = 18)	p-value
Age (years) <63 ≥63	40 (49.4) 41 (50.6)	32 (50.8) 31 (49.2)	8 (44.4) 10 (55.6)	0.64
Gender, n (%) Female Male	22 (27.2) 59 (72.8)	17 (27.0) 46 (73.0)	5 (27.8) 13 (72.2)	0.95
Histology, n (%) Clear cell carcinoma Non-clear cell carcinoma	68 (84) 13 (16)	53 (84.1) 10 (15.9)	15 (83.3) 3 (16.7)	0.94
Prior nephrectomy, n (%) No Yes	23 (28.4) 58 (71.6)	17 (27.0) 46 (73.0)	6 (33.3) 12 (66.7)	0.60
Nivolumab treatment line, n (%) Second-line Beyond second-line	39 (48.1) 42 (51.9)	27 (42.9) 36 (57.1)	12 (66.7) 6 (33.3)	0.08
ECOG performance score, n (%) ECOG 0 ECOG ≥1	50 (61.7) 31 (38.3)	42 (66.7) 21 (33.3)	8 (44.4) 10 (55.6)	0.09
IMDC Risk group, n (%) Favorable - Intermediate Poor	65 (80.2) 16 (19.8)	53 (84.1) 10 (15.9)	12 (66.7) 6 (33.3)	0.10
MSKCC Risk group, n (%) Good - Intermediate Poor	66 (81.5) 15 (18.5)	52 (82.5) 11 (17.5)	14 (77.8) 4 (22.2)	0.65
Lung metastasis, n (%) No Yes	14 (17.3) 67 (82.7)	8 (12.7) 55 (87.3)	6 (33.3) 12 (66.7)	**0.04**
Liver metastasis, n (%) No Yes	65 (80.2) 16 (19.8)	51 (81.0) 12 (19.0)	14 (77.8) 4 (22.2)	0.77
Bone metastasis, n (%) No Yes	43 (53.1) 38 (46.9)	35 (55.6) 28 (44.4)	8 (44.4) 10 (55.6)	0.41

ECOG, Eastern Cooperative Oncology Group; IMDC, International Metastatic RCC Database Consortium; MSKCC, Memorial Sloan Kettering Cancer Center; IQR, Interquartile Range. The bold value indicates statistically significant results (p < 0.05).

### Treatment responses and outcomes across the entire cohort and GRIm score groups objective response rates and duration of responses

Among all patients, the majority achieved disease control (DCR: 60.5%), with an overall objective response rate (ORR) of 37% (30/81). Detailed response outcomes, including CR, PR, and DCR, are presented in **Table 2**.

**Table 2:** Response evaluation and survival outcomes across the entire cohort and GRIm score groups.

Response and survival parameters	All patients (n=81)	Low group (n=63)	High group (n=18)	p value
Complete response, n (%)	7 (8.6)	6 (9.5)	1 (5.6)	**0.01**
Partial response, n (%)	23 (28.4)	22 (34.9)	1 (5.6)	
Stable disease, n (%)	19 (23.5)	14 (22.2)	5 (27.8)	
Progressive disease, n (%)	32 (39.5)	21 (33.3)	11 (61.1)	
Objective response rate, n (%)	30 (37.0)	28 (44.4)	2 (11.1)	**0.01**
Disease control rate, n (%)	49 (60.5)	42 (66.7)	7 (38.9)	**0.03**
Progression-free survival, months; Median (Lower – Upper)	7.3 (1.6-13.1)	8.7 (1.2-16.2)	3.1 (0.1-6.9)	**0.015**
Overall survival, months; Median (Lower – Upper)	19.6 (11.2-28.1)	23.3 (3.8-42.8)	8.8 (0.1-19.5)	**0.004**

The overall ORR for the entire cohort was 37% (30/81), while subgroup ORRs were 44.4% (28/63) in the low GRIm group and 11.1% (2/18) in the high GRIm group. The bold values indicate statistically significant results (p < 0.05).

In the low GRIm score group, response rates were as follows: CR: 9.5% (n = 6/63), PR: 34.9% (n = 22/63), yielding an ORR of 44.4% (n = 28/63).

In contrast, the high GRIm score group demonstrated markedly lower responses: CR: 5.6% (n = 1/18) and PR: 5.6% (n = 1/18), corresponding to an ORR of 11.1% (n = 2/18). The difference in ORR between the groups was statistically significant (p = 0.01).

Regarding response duration, in the low GRIm group, one CR patient experienced progression after 24 months, whereas the remaining CR patients showed sustained responses lasting 26–56 months at the data cutoff. Among PR patients, the median duration of response (DOR) was 18 months (95% CI: 1–37.5), with durable responses observed in 45.5% of cases (n = 10/22).

In the high GRIm group, the single CR patient had a response duration of 54 months, while the single PR patient experienced a shorter response duration of 11 months.

### Analysis for progression-free survival and for overall survival

**Figure 1:**
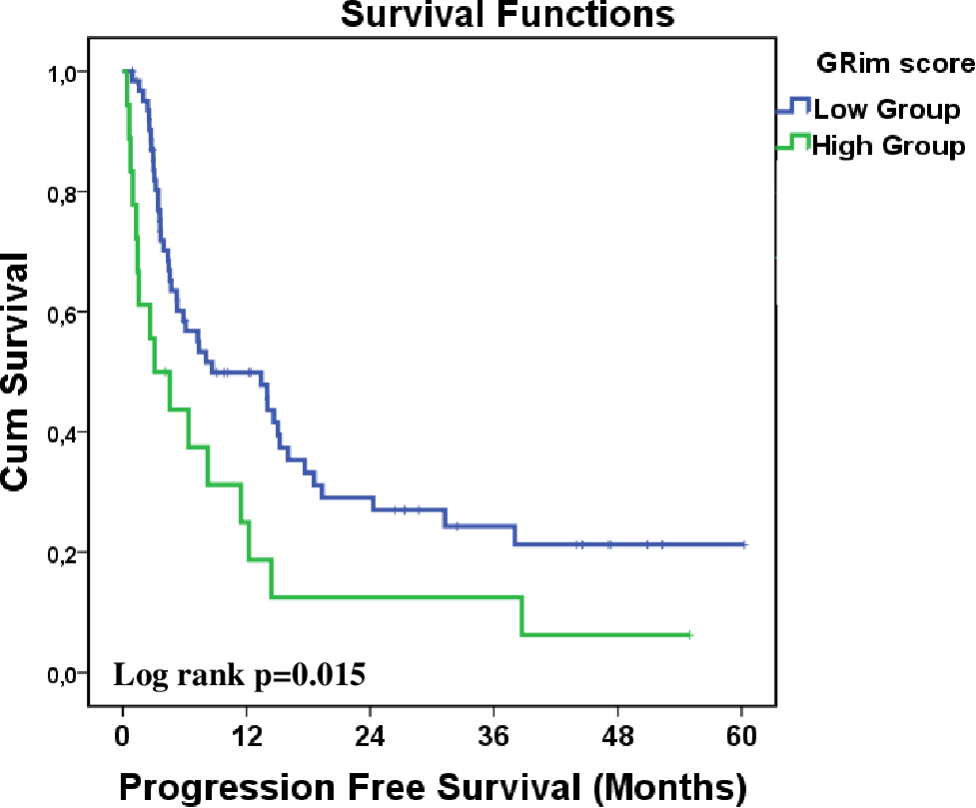
Kaplan–Meier analysis of progression-free survival stratified by GRIm score (low *vs*. high).

**Figure 2:**
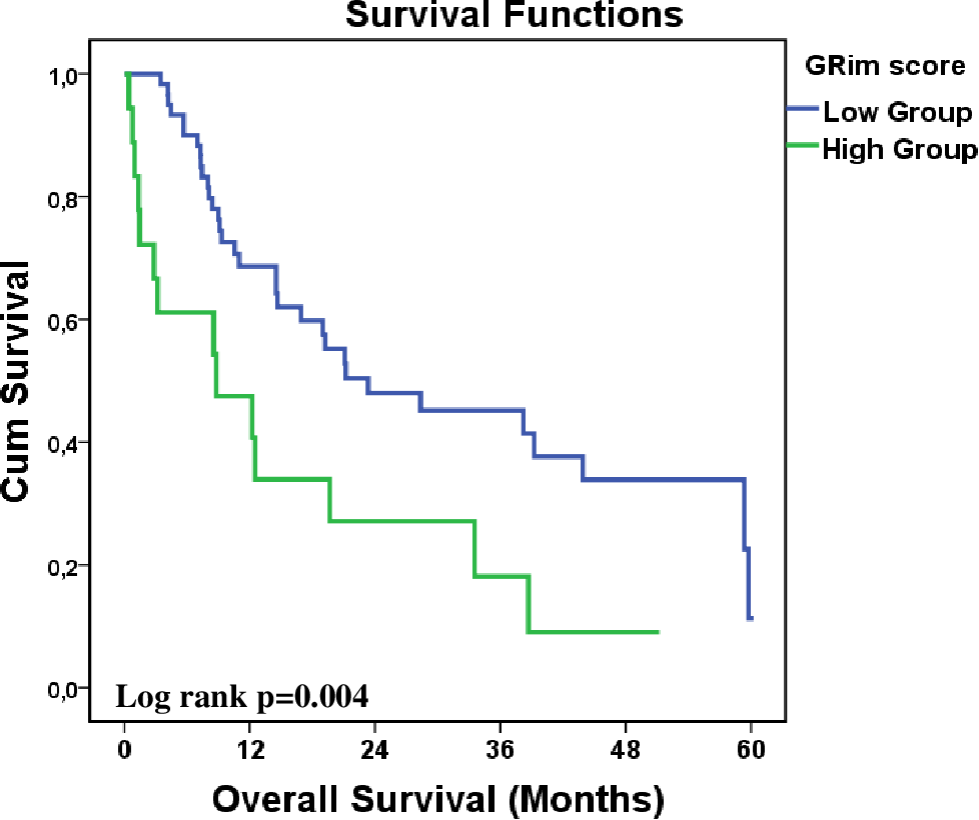
Kaplan–Meier analysis of overall survival stratified by GRIm score (low *vs*. high).

PFS was significantly longer in the low GRIm score group at 8.7 months (95% CI: 1.2–16.2) compared to 3.1 months (95% CI: 0.1–6.9) in the high GRIm score group (p = 0.015). Kaplan-Meier curves illustrating PFS are presented in **Figure 1**, highlighting this statistically significant difference between the groups. OS was also significantly longer in the low GRIm score group at 23.3 months (95% CI: 3.8–42.8) compared to 8.8 months (95% CI: 0.1–19.5) in the high GRIm score group (p = 0.004). The Kaplan-Meier curves for OS, shown in **Figure 2**, demonstrate a clear survival advantage for patients in the low GRIm score group.

In the univariate analysis for PFS, the GRIm score was identified as the only significant clinical parameter. Patients in the high GRIm score group had a significantly higher risk of disease progression or death compared to those in the low GRIm score group (HR: 2.01, 95% CI: 1.12–3.58, p = 0.017). No other clinical or demographic factors demonstrated significant associations with PFS.

In the multivariate analysis for PFS, the high GRIm score retained its status as an independent prognostic factor for worse progression-free survival (HR: 2.24, 95% CI: 1.29–4.59, p = 0.006). No other variables, including prior nephrectomy, IMDC risk group, or metastasis sites, reached statistical significance.

In the univariate analysis for OS, three clinical parameters were identified as significant or borderline significant prognostic factors. Patients without prior nephrectomy showed a significantly worse OS compared to those who had undergone the procedure (HR: 0.54, 95% CI: 0.29–0.99, p = 0.049). Patients in the high GRIm score group were associated with a markedly worse OS compared to those with a low GRIm score (HR: 0.41, 95% CI: 0.22–0.78, p = 0.006). The poor-risk group demonstrated borderline significance, with a trend toward worse OS compared to the low/intermediate group (HR: 1.76, 95% CI: 0.93–3.42, p = 0.09).

In the multivariate analysis for OS, the only independent prognostic factor associated with worse OS (HR: 2.17, 95% CI: 1.09–4.34, p = 0.03) was high GRIm score. Although variables including bone metastasis and lung metastasis were not significant in the univariate analysis, they were included in the multivariate model to ensure a comprehensive evaluation. However, these variables did not maintain statistical significance in the multivariate analysis (p > 0.05).

Both univariate and multivariate analyses for PFS and OS were presented in detail in **Table 3**, highlighting the significance of key clinical and prognostic factors.

**Table 3:** Univariate and multivariate analyses of clinical features for progression-free and overall survival.

	Progression-free survival		Overall survival analysis			
	Univariate analysis		Univariate analysis		Multivariate analysis	
	HR (95% CI, Lower-Upper)	P value	HR (95% CI, Lower-Upper)	P value	HR (95% CI, Lower-Upper)	P value
Age <63 vs ≥63 years	0.84 (0.5-1.4)	0.51	0.73 (0.45-1.36)	0.43		
Gender Female vs Male	0.94 (0.52-1.68)	0.83	1 (0.52-1.91)	0.98		
Histology Clear vs Non-Clear	1.22 (0.59-2.49)	0.58	1.4 (0.67-2.9)	0.37		
Prior nephrectomy Yes vs No	0.72 (0.41-1.27)	0.26	**0.54 (0.29-0.99)**	**0.049**	0.61 (0.31-1.23)	0.16
Treatment line Second vs Beyond S.	0.82 (0.49-1.36)	0.44	1.41 (0.78-2.52)	0.25		
ECOG 0 vs ≥1	1.14 (0.67-1.92)	0.63	1.02 (0.56-1.87)	0.94		
IMDC Risk group Favorable-Intermediate vs Poor	1.35 (0.73-2.5)	0.34	**1.76 (0.91-3.42)**	**0.09**	1.1 (0.48-2.45)	0.83
MSKCC Risk group Good-Intermediate vs Poor	1.1 (0.55-2.1)	0.81	1.4 (0.69-2.83)	0.35		
Lung metastasis No vs Yes	0.61 (0.32-1.19)	0.15	0.61 (0.30-1.23)	0.17	0.86 (0.39-1.91)	0.72
Liver metastasis No vs Yes	1.05 (0.55-1.99)	0.87	1.02 (0.50-2.06)	0.93		
Bone metastasis No vs Yes	1.15 (0.69-1.94)	0.57	1.5 (0.83-2.71)	0.17	1.11 (0.57-2.15)	0.77
GRIm Score Low vs High	**2.01 (1.12-3.58)**	**0.017**	**2.24 (1.29-4.59)**	**0.006**	**2.17 (1.09-4.34)**	**0.03**

ECOG, Eastern Cooperative Oncology Group; IMDC, International Metastatic RCC Database Consortium; MSKCC, Memorial Sloan Kettering Cancer Center; GRIm, Gustave Roussy Immune Score. The bold value indicates statistically significant results (p < 0.05).

## Discussion

Prompt identification of mRCC patients who are likely to benefit from nivolumab, a PD-1 inhibitor, is crucial in clinical practice. Effective patient stratification ensures optimized treatment outcomes and minimizes unnecessary toxicities. In this context, this paper is the first to evaluate the prognostic value of the GRIm score in mRCC patients undergoing nivolumab therapy. An analysis of 81 patients revealed that a high GRIm score was significantly associated with poor oncological outcomes, demonstrating its effectiveness as a tool for predicting treatment response. Findings in this paper are consistent with prior reports supporting the role of immune checkpoint inhibitors (ICIs) across various cancer types (13, 15–18).

In mRCC patients progressing during or after anti-angiogenic therapy, nivolumab has become a standard treatment option due to its demonstrated OS benefits, durable responses, and favorable safety profile. The phase III CheckMate 025 trial enrolled 821 patients with advanced mRCC who had received one or two prior anti-angiogenic therapies. In this trial, nivolumab achieved a median OS of 25.8 months (95% CI: 22.4–30.0), a median PFS of 4.6 months (95% CI: 3.7–5.4), and an ORR of 23 percent, including 1 percent CR and 22 percent PR. Furthermore, nivolumab demonstrated fewer grade 3 or higher adverse events (21 percent versus 37 percent) and significant improvements in quality of life (5, 19). In our real-world mRCC cohort treated with nivolumab, the median OS was 19.6 months (95% CI: 11.0–28.1), and the median PFS was 7.4 months (95% CI: 1.7–13.1). The ORR was 37%, including 8.6% CR and 28.4% PR. The relatively high CR rate may explain the longer median PFS and suggest greater treatment efficacy in certain patient subgroups. The shorter OS may be related to shorter follow-up durations, worse ECOG performance status (≥1), and the use of nivolumab in later lines of therapy. However, adverse events were not systematically documented in this retrospective cohort, and therefore a comparative analysis of toxicity between GRIm score groups could not be performed. This represents an additional limitation of the study.

Despite its efficacy, nivolumab does not benefit all patients equally, highlighting the need for reliable biomarkers to better stratify patients and optimize treatment strategies. Biomarkers such as tumor mutational burden (TMB) and PD-L1 expression, while predictive in other cancers, have limited utility in mRCC. In mRCC, PD-L1 expression is associated with aggressive tumor phenotypes but has not consistently predicted response to immunotherapy. Similarly, TMB levels are generally low in mRCC and show weak associations with immunotherapy efficacy (20, 21). Instead, the effectiveness of immunotherapy in mRCC appears to be more influenced by the tumor’s immunological microenvironment and genetic characteristics. Factors such as CD8+ T-cell infiltration, tumor-associated macrophage polarization, and the presence of proinflammatory cytokines have emerged as more robust predictors of response. Understanding these elements and integrating them into clinical practice could improve patient selection and optimize outcomes in mRCC (21, 22).

Inflammation and nutritional deficiencies are key factors impacting cancer progression, treatment response, and prognosis. Peripheral blood-based biomarkers have gained attention for their ability to reflect these parameters and assist in patient stratification. When used in combination, these markers have shown greater utility in predicting survival outcomes and guiding treatment strategies (9–12). In this context, the GRIm score was developed in 2017 during phase I immunotherapy trials to improve patient selection and predict survival beyond three months. Designed to address the limitations of the Royal Marsden Hospital (RMH) score in immunotherapy settings, the GRIm score incorporates albumin, LDH, and NLR to provide a comprehensive prognostic tool (13).

The GRIm score has been validated across various cancers, demonstrating significant prognostic value. A meta-analysis of 15 studies (20 cohorts) including 4997 cancer patients showed that high GRIm scores were strongly associated with worse OS (HR = 2.07; 95% CI: 1.73–2.48; p < 0.0001; I^2^ = 62%) and shorter PFS (HR = 1.42; 95% CI: 1.22–1.66; p < 0.0001; I^2^ = 36%) (14). These findings highlight its utility in clinical decision-making and its potential to optimize outcomes in diverse patient populations.

Our study is the first to evaluate the prognostic value of the GRIm score in mRCC patients treated with nivolumab. Stratification using the GRIm score has been shown to be a valuable tool in predicting treatment response rates and survival outcomes. Median OS was calculated as 23.3 months in patients with low GRIm scores, compared to only 8.8 months in those with high GRIm scores (p = 0.004). Similarly, median PFS was 8.7 months (95% CI: 1.2–16.2) in the low GRIm group, while it was 3.1 months (95% CI: 0.1–6.9) in the high GRIm group (p = 0.015).

Notably, PFS in patients who achieved PR in the low GRIm group was calculated as 18 months (95% CI: 1–37.5), more than twice the PFS observed in the overall group (7.3 months). This finding highlights the dramatic improvement in progression-free survival among patients with low GRIm scores who respond to treatment. Furthermore, sustained response was observed in 45.5% of PR patients (n = 10/22), reinforcing the importance of the GRIm score in selecting appropriate patients for long-term disease control.

Additionally, most patients who achieved CR in the low GRIm group did not experience progression, with response durations ranging between 26 and 56 months. In contrast, both CR and PR rates were limited to 5.6% in the high GRIm group, indicating a lower likelihood of treatment response in these patients. The GRIm score effectively identifies this low-response subgroup early, enabling the consideration of alternative therapeutic strategies. Multivariate analysis identified a high GRIm score as an independent negative prognostic factor for OS (HR: 0.46; p = 0.03). Moreover, high GRIm scores were strongly associated with poor ECOG performance status (≥1) and later lines of nivolumab treatment.

The GRIm score derives its efficacy from three pivotal biomarkers: LDH, NLR, and albumin, each contributing uniquely to tumor progression and patient prognosis (13). LDH supports tumor growth and metastasis by meeting energy demands and promoting an inflammatory microenvironment through IL-17 and IL-23 activation. This process suppresses CD8+ T lymphocytes and natural killer cells, facilitating immune evasion (23). NLR reflects the pro-tumor activity of neutrophils and the immunosuppressive effects of reduced lymphocyte counts, driving tumor progression (24, 25). Albumin, a marker of nutritional status, is associated with key outcomes such as wound healing, infection risk, and survival, with hypoalbuminemia exacerbating inflammation and immune dysfunction (26, 27). Evidence from RCC studies validates the prognostic value of these biomarkers. Meta-analyses confirm their strong correlation with adverse survival outcomes, reinforcing their clinical relevance (28–30).

Recent advances in bladder cancer research illustrate how rational drug design and biomarker-driven strategies are transforming the therapeutic landscape of urinary system tumors. A large multicenter real-world study evaluating disitamab vedotin (RC48-ADC) in combination with immunotherapy demonstrated remarkable pathological response rates, clearly outperforming historical chemotherapy-based regimens. Importantly, this trial incorporated exploratory biomarker analyses that linked treatment sensitivity to specific immune microenvironmental features, highlighting the dual clinical and translational value of such integrative approaches (31). Building on these findings, retrospective multicenter data comparing immunotherapy, chemotherapy, and their combinations in the neoadjuvant setting confirmed that rationally designed combination strategies yield superior pathological responses and prolonged survival compared with monotherapy. The results emphasize the heterogeneity of patient subgroups, underscoring that therapeutic benefit is not uniform and must be interpreted within the context of biological diversity. This reinforces the need for predictive biomarkers that can guide regimen selection, ensuring that each patient receives the therapy most aligned with their tumor biology (32). More recently, the concept of bladder preservation has emerged as a feasible outcome for selected patients treated with neoadjuvant immunotherapy, as demonstrated by propensity score–matched multicenter analyses. Notably, this work integrated advanced multi-omics techniques, including single-cell RNA sequencing, which uncovered specific stromal and immune signatures associated with durable disease control. These molecular insights provide mechanistic explanations for clinical outcomes and exemplify how multi-omics exploration can refine patient selection, moving beyond traditional clinicopathological parameters (33). Collectively, these studies highlight how combining novel agents, rational treatment sequencing, and multi-omics biomarker discovery can advance both survival outcomes and organ preservation strategies, setting a new standard for the management of urinary system tumors.

The GRIm score integrates accessible and clinically relevant biomarkers to enhance patient stratification and guide tailored treatment strategies. Its incorporation into clinical practice can optimize patient selection, improve therapeutic efficacy, and minimize unnecessary toxicities, offering a pragmatic approach to advancing cancer care in real-world settings.

This study, while the first to evaluate the prognostic significance of the GRIm score in RCC patients treated with nivolumab, has several limitations. Its multicenter, retrospective design and relatively small sample size limit the generalizability of the findings. Additionally, the study did not assess other inflammation- or nutrition-based prognostic markers such as NLR, dNLR, PLR, LMR, or SII, which could provide a more comprehensive analysis. Equally important, the lack of longitudinal GRIm score measurements limited our ability to evaluate dynamic changes during the course of treatment. Such temporal assessments could have provided valuable insight into whether fluctuations in the GRIm score correlate with evolving treatment efficacy or emerging resistance, thereby refining its prognostic strength, particularly for overall survival. Lastly, while nivolumab was uniformly used in this cohort, variations in prior TKI treatments and other patient-specific factors may have contributed to the heterogeneity of responses. These limitations highlight the need for larger, multicenter, prospective studies to validate the findings and refine the GRIm score’s clinical utility.

Beyond these constraints, several additional limitations should be acknowledged. First, adverse events were not systematically documented in this retrospective analysis, which precluded meaningful comparison of toxicity profiles across GRIm score groups. Since safety outcomes represent a key determinant of treatment continuation and quality of life, the absence of these data limits the comprehensiveness of our evaluation. Second, the retrospective design carries inherent risks of selection bias and incomplete data capture, particularly with respect to baseline characteristics such as comorbidities, nutritional status, and prior treatment tolerability, all of which may influence survival outcomes. Third, the relatively short and heterogeneous follow-up period may have led to an underestimation of long-term survival outcomes, especially for patients achieving durable responses. Moreover, while nivolumab was uniformly applied in this cohort, variations in prior targeted therapies and line of treatment may have confounded survival outcomes, reducing the precision of our estimates. Finally, the absence of external validation and biomarker integration, such as PD-L1 expression, tumor mutational burden, or multi-omics profiling, prevents extrapolation of the GRIm score’s predictive value beyond the studied population. Collectively, these limitations highlight the need for larger, multicenter, prospective studies with standardized toxicity reporting and integration of molecular and immunological biomarkers to confirm and extend our findings.

## Conclusion

This study highlights the potential of the GRIm score as a simple, cost-effective prognostic tool for patients with metastatic RCC treated with nivolumab in second-line or subsequent settings. The findings demonstrate that a high GRIm score is independently associated with worse survival and treatment response, emphasizing its utility in patient stratification. While further multicenter, prospective studies are needed to validate and expand these results, the GRIm score offers a promising avenue for optimizing treatment decisions and improving outcomes in clinical practice. In addition, incorporating the GRIm score into prospective trials together with emerging molecular and multi-omics biomarkers may further refine patient selection strategies and ultimately contribute to more personalized therapeutic approaches in metastatic RCC.

## Data Availability

The original contributions presented in the study are included in the article/**Supplementary Material**. Further inquiries can be directed to the corresponding author.

## Supplementary material

SUPPLEMENTARY FIGURE 1 Schematic illustration of the Gustave Roussy Immune (GRIm) score and its prognostic implications in advanced renal cell carcinoma.

SUPPLEMENTARY FIGURE 2 Kaplan–Meier survival curves showing progression-free survival and overall survival according to GRIm score groups. Both figures are original, contain no copyrighted or identifiable material, and require no further changes.

## References

[1] Bukavina L Bensalah K Bray F Carlo M Challacombe B Karam JA. Epidemiology of renal cell carcinoma: 2022 update. Eur Urol. (2022) 82:529–42. doi: 10.1016/j.eururo.2022.08.019, PMID: 36100483

[2] Chen DS Mellman I. Oncology meets immunology: the cancer-immunity cycle. Immunity. (2013) 39:1–10. doi: 10.1016/j.immuni.2013.07.012, PMID: 23890059

[3] de Velasco G Bex A Albiges L Powles T Rini BI Motzer RJ. Sequencing and combination of systemic therapy in metastatic renal cell carcinoma. Eur Urol Oncol. (2019) 2:505–14. doi: 10.1016/j.euo.2019.06.022, PMID: 31377308

[4] Garje R An J Greco A Vaddepally RK Zakharia Y. The future of immunotherapy-based combination therapy in metastatic renal cell carcinoma. Cancers (Basel). (2020) 12:123. doi: 10.3390/cancers12010143, PMID: 31936065 PMC7017064

[5] Motzer RJ Escudier B George S Hammers HJ Srinivas S Tykodi SS. Nivolumab versus everolimus in patients with advanced renal cell carcinoma: updated results with long-term follow-up of the randomized, open-label, phase 3 CheckMate 025 trial. Cancer. (2020) 126:4156–67. doi: 10.1002/cncr.33033, PMID: 32673417 PMC8415096

[6] Ljungberg B Albiges L Abu-Ghanem Y Bedke J Capitanio U Dabestani S. European Association of Urology guidelines on renal cell carcinoma: the 2022 update. Eur Urol. (2022) 82:399–410. doi: 10.1016/j.eururo.2022.03.006, PMID: 35346519

[7] Heng DY Xie W Regan MM Harshman LC Bjarnason GA Vaishampayan UN. External validation and comparison with other models of the International Metastatic Renal-Cell Carcinoma Database Consortium prognostic model: a population-based study. Lancet Oncol. (2013) 14:141–8. doi: 10.1016/S1470-2045(12)70559-4, PMID: 23312463 PMC4144042

[8] Motzer RJ Bacik J Murphy BA Russo P Mazumdar M. Interferon-alfa as a comparative treatment for clinical trials of new therapies against advanced renal cell carcinoma. J Clin Oncol. (2002) 20:289–96. doi: 10.1200/JCO.2002.20.1.289, PMID: 11773181

[9] Nishiyama N Hirobe M Kikushima T Matsuki M Takahashi A Yanase M. The neutrophil-lymphocyte ratio has a role in predicting the effectiveness of nivolumab in Japanese patients with metastatic renal cell carcinoma: a multi-institutional retrospective study. BMC Urol. (2020) 20:110. doi: 10.1186/s12894-020-00679-2, PMID: 32711491 PMC7382809

[10] Rebuzzi SE Signori A Banna GL Maruzzo M De Giorgi U Pedrazzoli P. Inflammatory indices and clinical factors in metastatic renal cell carcinoma patients treated with nivolumab: the development of a novel prognostic score (Meet-URO 15 study). Ther Adv Med Oncol. (2021) 13:17588359211019642. doi: 10.1177/17588359211019642, PMID: 34046089 PMC8135208

[11] De Giorgi U Procopio G Giannarelli D Sabbatini R Bearz A Buti S. Association of systemic inflammation index and body mass index with survival in patients with renal cell cancer treated with nivolumab. Clin Cancer Res. (2019) 25:3839–46. doi: 10.1158/1078-0432.CCR-18-3661, PMID: 30967420

[12] Nakayama T Takeshita H Kagawa M Washino S Shirotake S Miura Y. Prognostic significance of inflammatory markers in patients with advanced renal cell carcinoma receiving nivolumab plus ipilimumab. Int J Clin Oncol. (2024) 29:1528–37. doi: 10.1007/s10147-024-02593-1, PMID: 39046676

[13] Bigot F Castanon E Baldini C Hollebecque A Carmona A Postel-Vinay S. Prospective validation of a prognostic score for patients in immunotherapy phase I trials: the Gustave Roussy Immune Score (GRIm-score). Eur J Cancer. (2017) 84:212–8. doi: 10.1016/j.ejca.2017.07.027, PMID: 28826074

[14] Sun KX Xu RQ Rong H Pang HY Xiang TX. Prognostic significance of the Gustave Roussy Immune (GRIm) score in cancer patients: a meta-analysis. Ann Med. (2023) 55:2236640. doi: 10.1080/07853890.2023.2236640, PMID: 37851510 PMC10586078

[15] Kitadai R Okuma Y Hakozaki T Hosomi Y. The efficacy of immune checkpoint inhibitors in advanced non-small-cell lung cancer with liver metastases. J Cancer Res Clin Oncol. (2020) 146:777–85. doi: 10.1007/s00432-019-03104-w, PMID: 31828427 PMC11804625

[16] Li Y Pan Y Lin X Hou J Hu Z Xu L. Development and validation of a prognostic score for hepatocellular carcinoma patients in immune checkpoint inhibitor therapies: the hepatocellular carcinoma modified Gustave Roussy Immune Score. Front Pharmacol. (2021) 12:819985. doi: 10.3389/fphar.2021.819985, PMID: 35237150 PMC8883391

[17] Nakazawa N Sohda M Ubukata Y Kuriyama K Kimura A Kogure N. Changes in the Gustave Roussy Immune Score as a powerful prognostic marker of the therapeutic sensitivity of nivolumab in advanced gastric cancer: a multicenter, retrospective study. Ann Surg Oncol. (2022) 29:7400–6. doi: 10.1245/s10434-022-12226-4, PMID: 35857197

[18] Minichsdorfer C Gleiss A Aretin MB Schmidinger M Fuereder T. Serum parameters as prognostic biomarkers in a real-world cancer patient population treated with anti-PD-1/PD-L1 therapy. Ann Med. (2022) 54:1339–49. doi: 10.1080/07853890.2022.2070660, PMID: 35535695 PMC9103267

[19] Motzer RJ Escudier B McDermott DF George S Hammers HJ Srinivas S. Nivolumab versus everolimus in advanced renal-cell carcinoma. N Engl J Med. (2015) 373:1803–13. doi: 10.1056/NEJMoa1510665, PMID: 26406148 PMC5719487

[20] Jani Y Jansen CS Gerke MB Bilen MA. Established and emerging biomarkers of immunotherapy in renal cell carcinoma. Immunotherapy. (2024) 16:405–26. doi: 10.2217/imt-2023-0267, PMID: 38264827 PMC11913054

[21] Brown LC Zhu J Desai K Kinsey E Kao C Lee YH. Evaluation of tumor microenvironment and biomarkers of immune checkpoint inhibitor response in metastatic renal cell carcinoma. J Immunother Cancer. (2022) 10:e004567. doi: 10.1136/jitc-2022-005249, PMID: 36252996 PMC9577926

[22] Xu X Lin J Wang J Wang Y Zhu Y Wang J. SPP1 expression indicates outcome of immunotherapy plus tyrosine kinase inhibition in advanced renal cell carcinoma. Hum Vaccin Immunother. (2024) 20:2350101. doi: 10.1080/21645515.2024.2350101, PMID: 38738709 PMC11093034

[23] Ding J Karp JE Emadi A. Elevated lactate dehydrogenase (LDH) can be a marker of immune suppression in cancer: interplay between hematologic and solid neoplastic clones and their microenvironments. Cancer biomark. (2017) 19:353–63. doi: 10.3233/CBM-160336, PMID: 28582845 PMC13020749

[24] Li Y Xu T Wang X Jia X Ren M Wang X. The prognostic utility of preoperative neutrophil-to-lymphocyte ratio (NLR) in patients with colorectal liver metastasis: a systematic review and meta-analysis. Cancer Cell Int. (2023) 23:39. doi: 10.1186/s12935-023-02876-z, PMID: 36855112 PMC9976405

[25] Melssen MM Sheybani ND Leick KM Slingluff CL Jr. Barriers to immune cell infiltration in tumors. J Immunother Cancer. (2023) 11:e006789. doi: 10.1136/jitc-2022-006401, PMID: 37072352 PMC10124321

[26] Gupta D Lis CG. Pretreatment serum albumin as a predictor of cancer survival: a systematic review of the epidemiological literature. Nutr J. (2010) 9:69. doi: 10.1186/1475-2891-9-69, PMID: 21176210 PMC3019132

[27] Navaei-Alipour N Mastali M Ferns GA Saberi-Karimian M Ghayour-Mobarhan M. The effects of honey on pro- and anti-inflammatory cytokines: a narrative review. Phytother Res. (2021) 35:3690–701. doi: 10.1002/ptr.7066, PMID: 33751689

[28] Shen J Chen Z Zhuang Q Fan M Ding T Lu H. Prognostic value of serum lactate dehydrogenase in renal cell carcinoma: a systematic review and meta-analysis. PloS One. (2016) 11:e0166482. doi: 10.1371/journal.pone.0166482, PMID: 27861542 PMC5115746

[29] Zhou X Fu G Zu X Xu Z Li HT D’Souza A. Albumin levels predict prognosis in advanced renal cell carcinoma treated with tyrosine kinase inhibitors: a systematic review and meta-analysis. Urol Oncol. (2022) 40:12.e3–12.e22. doi: 10.1016/j.urolonc.2021.08.001, PMID: 34454823

[30] Hu K Lou L Ye J Zhang S. Prognostic role of the neutrophil-lymphocyte ratio in renal cell carcinoma: a meta-analysis. BMJ Open. (2015) 5:e006404. doi: 10.1136/bmjopen-2014-006404, PMID: 25854964 PMC4390726

[31] Hu J Yan L Liu J Chen M Liu P Deng D. Efficacy and biomarker analysis of neoadjuvant disitamab vedotin (RC48-ADC) combined with immunotherapy in patients with muscle-invasive bladder cancer: A multi-center real-world study. iMeta. (2025) 4(3):e70033. doi: 10.1002/imt2.70033, PMID: 40469503 PMC12130573

[32] Hu J Chen J Ou Z Chen H Liu Z Chen M. Neoadjuvant immunotherapy, chemotherapy, and combination therapy in muscle-invasive bladder cancer: A multi-center real-world retrospective study. Cell Rep Med. (2022) 3:100785. doi: 10.1016/j.xcrm.2022.100785, PMID: 36265483 PMC9729796

[33] Hu J Yan L Liu J Chen M He Y Fan B. Neoadjuvant immunotherapy-driven bladder preservation for muscle-invasive bladder cancer: A multicenter study. iMeta. (2025) 4(4):e70063. doi: 10.1002/imt2.70063

