# Supplementary figures and images for: Gustave Roussy immune score as an independent prognostic factor for treatment response and survival in advanced renal cell carcinoma treated with nivolumab in second-line and beyond
Source: Front Oncol. 2025 Oct 16;15:1657053. doi: 10.3389/fonc.2025.1657053 (PMC12571565; doi:10.3389/fonc.2025.1657053)

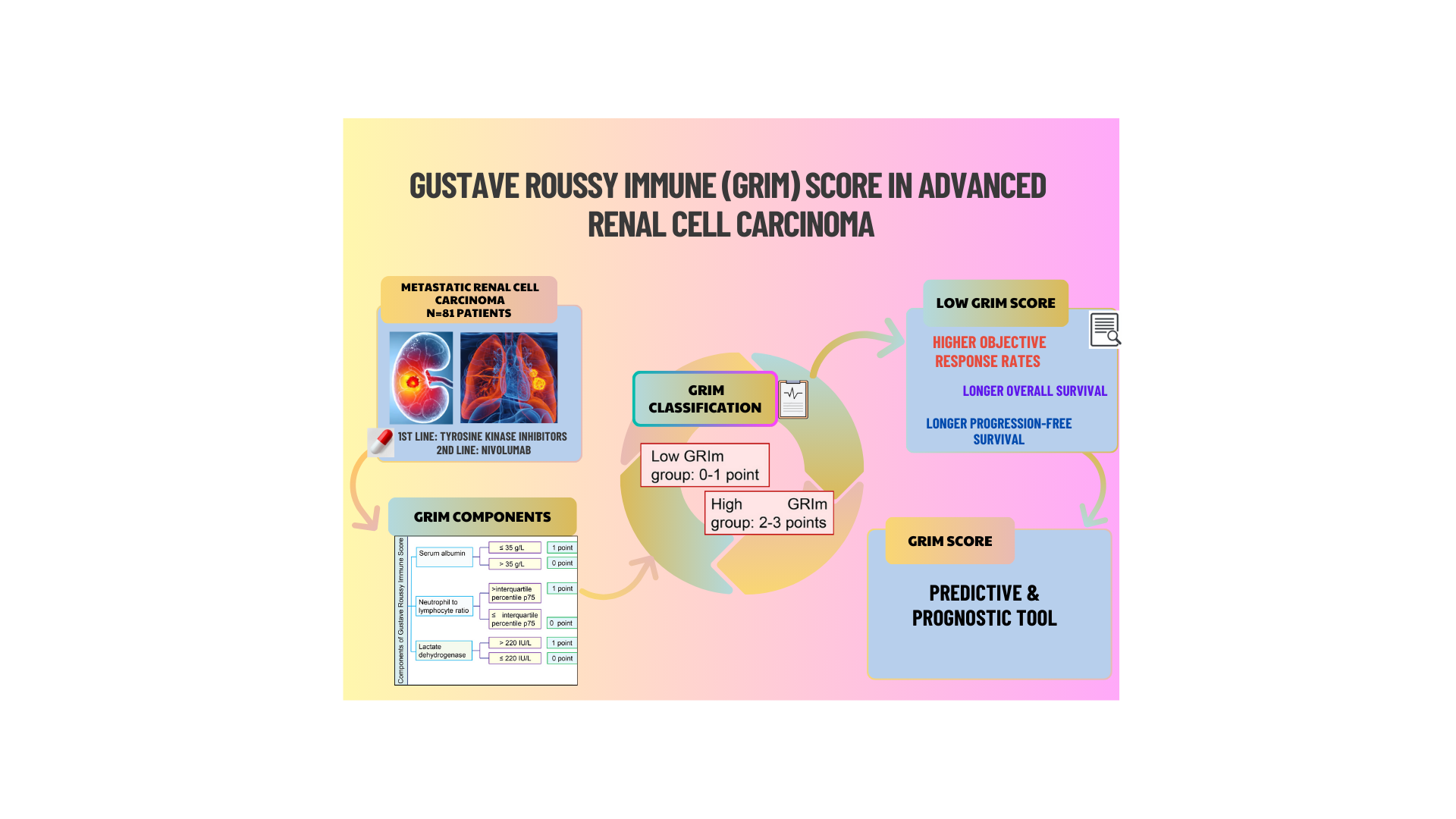

Supplement: SUPPLEMENTARY FIGURE 1 — Schematic illustration of the Gustave Roussy Immune (GRIm) score and its prognostic implications in advanced renal cell carcinoma. [file Image1.png]

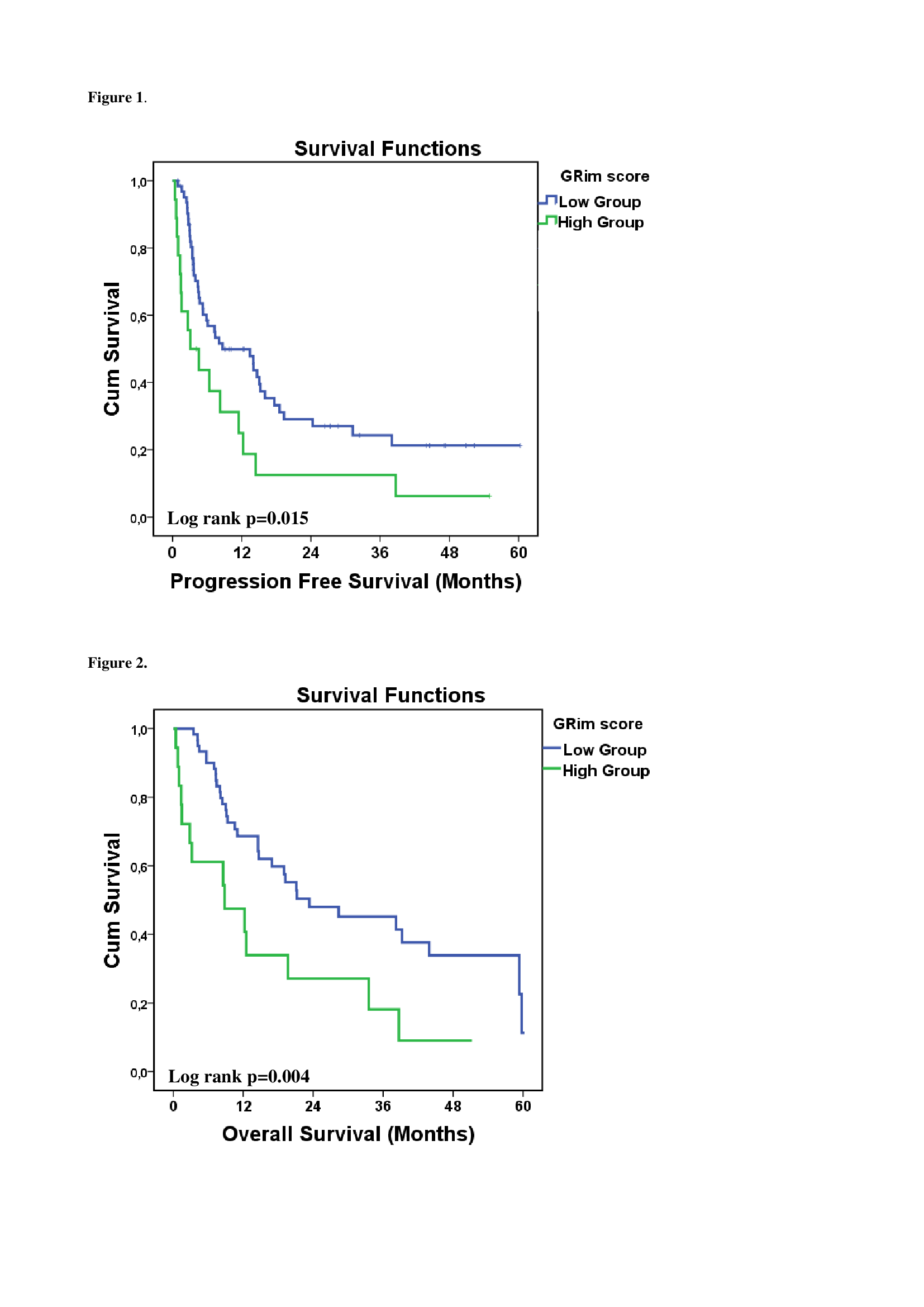

Supplement: SUPPLEMENTARY FIGURE 2 — Kaplan–Meier survival curves showing progression-free survival and overall survival according to GRIm score groups. Both figures are original, contain no copyrighted or identifiable material, and require no further changes. [file Image2.png]
